# Migraine With Comorbid Depression: Pathogenesis, Clinical Implications, and Treatment

**DOI:** 10.7759/cureus.25998

**Published:** 2022-06-16

**Authors:** Nailah Asif, Apurva Patel, Deepanjali Vedantam, Devyani S Poman, Lakshya Motwani

**Affiliations:** 1 Research, Ras Al Khaimah (RAK) College of Medical Sciences, Ras Al Khaimah, ARE; 2 Research, Gujarat Medical Education & Research Society (GMERS) Medical College, Vadodara, IND; 3 Internal Medicine, Kamineni Academy of Medical Sciences & Research Centre, Hyderabad, IND; 4 Research, Smolensk State Medical University, Smolensk, RUS; 5 Research and Development, Smt. Nathiba Hargovandas Lakhmichand (NHL) Municipal Medical College, Ahmedabad, IND

**Keywords:** migraine and psychiatric disorders, anti-cgrp monoclonal antibody, antidepressants in migraine, medication overuse headache, chronic migraine, fremanezumab, depression, migraine, migraine with depression

## Abstract

Migraine is a neurological disorder that strongly relates to psychiatric conditions like depression. Lately, the increased prevalence of depression in migraineurs has come to attention. This article compiled various literature to explore the association between migraine and depression. Genetic overlap of various gene segments was studied, and heritability patterns were explored. Shared mechanisms such as serotonergic dysfunction, methylenetetrahydrofolate reductase (MTHFR) polymorphisms, and hormonal effects were investigated, and commonalities like comorbidities, stress, and environmental factors were analyzed. Migraine with comorbid depression (MID) affects various aspects of life and its clinical impact on migraine disability, quality of life (QOL), progression, and medication overuse was investigated. We further inspected several types of research in order to provide options on various treatment modalities. Pharmacotherapy such as antidepressants and anti-Calcitonin Gene-Related Peptide (CGRP) monoclonal antibodies like fremanezumab were studied. Alternative treatment options such as onabotulinumtoxinA (OBTA) injections, cognitive behavioral therapy (CBT), and vagal nerve stimulations (VNS) were also appraised and the efficacies of each were compared.

## Introduction and background

Migraine is a chronic neurological disorder that is highly prevalent in society. It affects more than 10% of the world's population. Females are three times more likely than males to be diagnosed, and they account for 17.1% of the United States population. In contrast, males constitute only 5.6% of the population [1]. In 2019, it was the number one cause of years lived with disability in young women and came second for years lived with disability [2].

Migraine is characterized by a throbbing headache that may be associated with nausea, vomiting, photophobia, and phonophobia. Some are accompanied by an aura which usually manifests as visual symptoms such as flashing lights and haphazard lines (Table 1) [1]. The etiology is multifactorial, with several components playing a role. Genetics and environmental factors have been the major implications. Other aspects such as hormones, sleep, and psychological components may also influence the development of migraine [3].

**Table 1:** Diagnostic criterion of migraine

INTERNATIONAL HEADACHE SOCIETY CRITERIA
At least 5 or more attacks in a lifetime
Headache attack lasting 4-72 hours
At least 2 out of 4:
Unilateral location
Pulsating or throbbing quality
Moderate-severe intensity
Aggravation by/causing avoidance of routine physical activity
At least 1 of the following:
Nausea or vomiting
Photophobia
Phonophobia

Migraine can be classified into two types: episodic migraine (EM) and chronic migraine (CM). EM is defined as <15 headaches per month, while CM is defined as ≥15 headaches per month [4]. Because the transition from EM to CM is confined to a subset of the population, studying the risk factors might help with migraine management and prevention [5]. Risk factors of chronic migraine include high baseline attack frequency, medication overuse, obesity, snoring, caffeine consumption, and unsatisfactory treatment of migraine [5]. Other associated risk factors are female gender, anxiety, depression, allodynia, low socio-economic background, and comorbid pain disorders [6]. Various therapies have been used, ranging from medications to cognitive behavioral therapy [7]. However, its link to psychiatric comorbidities such as depression should be explored and targeted to improve quality of life and decrease the frequency, progression, and associated debilitation.

Major depressive disorder (MDD) is a psychiatric condition that has become increasingly common worldwide. In 2021, according to the Global Health Data Exchange, an estimated 3.8% of the world population had depression [8]. Like migraine, MDD is diagnosed more often in females than males [9] and is a major burden on society.

Numerous studies have demonstrated a strong link between migraine and depression [10]. A study done by Buse et al. demonstrated that those with CM were twice at risk of having depression [11]. The relationship between these two disorders is bidirectional; that is, an increased prevalence of depression has been found in migraineurs and vice versa [11, 12]. The process behind the association can be attributed to shared biological mechanisms, such as abnormal brain development and even genetic factors [13]. Comorbid depression complicates migraine diagnosis, and it also interferes with treatment compliance and may lead to overuse or abuse of medication. Furthermore, it may contribute to the progression of EM to CM [11, 13]. This may result in higher expenditures associated with the treatment along with inadequate management of the disease, ultimately culminating in increased migraine-related disability [13].

This review aims to explore the relationship between migraine and depression, highlight the effects of depression on migraine and suggest possible treatment options.

## Review

Shared pathogenesis

Depression is a common comorbidity of migraine. Several theories have been conjectured as to the possible mechanism behind this association.

Heritability

Prior research has shown that depression is more likely to develop in family members of those with migraine and vice versa [14]. Various studies on siblings and twins have found that genetic factors may play an essential role in the occurrence of depression in migraineurs [15, 16].

In Taiwan, a logistic regression analysis was done by Chen et al. amongst 1504 migraineurs, their 1595 unaffected siblings, and 6830 non-migrainous controls. Those who developed new-onset depression from 1996 to 2011 were identified. It was found that those who had migraine had an odds ratio (OR) of 4.09 (95% confidence interval (CI): 3.75-4.46) of developing depression, whereas the odds of developing depression in unaffected siblings was 1.40 (95% CI: 1.24-1.58) indicating that there is familial coaggregation of these conditions demonstrating that there was a statistically significant increased development of depression in siblings of those affected by migraine [14].

Another study done in Australia by Yang et al. in 5319 twins found that the co-twins of probands reporting any migraine had a significantly increased relative risk (RR) (RR = 1.12, 95% CI: 1.05-1.20) for depression. This association was more significant in monozygotic twins with an RR of 1.26 (95% CI: 1.14-1.38) [15].

Schur et al. obtained similar results in a study done amongst twins (758 monozygotic and 306 dizygotic female pairs). Of these, 23% reported depression and 20% reported migraines. Heritability was 58% (95% confidence interval: 48-67%) and 44% (95% confidence interval: 32-56%) for depression and migraine, respectively. It was estimated that 20% of the variability in depression and migraine was due to shared genetic factors [16].

Genetic Overlap

It has been determined that shared genetically determined biological mechanisms underlie both migraine and MDD. Several genome-wide association studies (GWAS) have been performed over the years for migraine and depression.

In 2018, a study done by Yang et al. showed significant genetic overlap in the loci identified for migraine and major depressive disorder (MDD). They used single-nucleotide polymorphism (SNP) and gene-based analysis of GWAS genotype data to conduct this study. Three SNPs (rs146377178, rs672931, and rs11858956) were implicated in the meta-analysis of 8,045,569 SNPs from a migraine GWAS (comprising 30,465 migraine cases and 143,147 control samples), and the top 10,000 SNPs from an MDD GWAS (comprising 75,607 MDD cases and 231,747 healthy controls). Gene-based analyses revealed significant enrichment of genes usually associated with migraine and MDD. Furthermore, two genes, ANKDD1B and KCNK5, produced Fisher’s combined gene-based P values that surpassed the genome-wide significance threshold (P_Fisher’s-combined_ ≤ 3.6 × 10^−6^) [17].

The Brainstorm Consortium was a collaboration among GWAS meta-analysis consortia of 25 brain disorders. They found a significant correlation between migraine and MDD (rg = 0.32, P = 1.42 × 10^−22^ for all migraine; rg = 0.23, P = 5.23 × 10^−5^ for migraine without aura; rg = 0.28, P = 1.00 × 10^−4^ for migraine with aura) [18].

A study done by Petschner et al. proved that SNPs of the previously identified migraine risk genes REST, ADGRL2, HPSE2 and 1p31.1 show lifetime depression-dependent associations with the disease [19].

Chen and Wang performed a study in 2020 based on microRNA (miRNA) biomarkers to investigate the association between migraine and depression. They discovered that 11 of the 12 migraine miRNA biomarkers were related to MDD [20].

A study done by Ligthart et al. in the Netherlands suggested that MID may be a completely different genetic disorder compared to isolated migraine. It involved a genetic risk score analysis in two sample groups in whom genome-wide association (GWA) analyses were performed. The first group, known as the Australian Twin Migraine GWA study (n=6350), consisted of 2825 migraineurs and 3525 controls, 805 of whom had MDD. The second group was the RADIANT GWA study (n=3230) which included 1636 MDD cases and 1594 controls. Genetic risk score analysis for migraine and MDD predicted pure and mixed forms of migraine and MDD. It was concluded that pure migraine and MDD were genetically distinct and that MID was genetically different from pure migraine. Moreover, MID was genetically closest in similarity to MDD [21].

Serotonergic Dysfunction

A defective serotonergic system has been linked to both depression and migraine. Low serotonin levels have been implicated in cortical spreading depression variance, influencing migraine risk [22]. The serotonin transporter-linked polymorphic region (5HTTLPR) of the serotonin transporter gene SLC6A4, in particular, has been considered a significant role player in the serotonergic system. Genetic polymorphisms of the serotonin gene (5HTPLLR) have also been extensively studied and are considered to contribute to migraine development [23-25].

Ren et al. in 2017 demonstrated low serotonin levels in migraineurs [26]. This was consistent with previous studies such as the one done by Ferrari et al. that found decreased plasma serotonin levels in between migraine episodes [27]. Similarly, Rossi et al. found significantly reduced serotonin levels in a cohort of CM patients [28]. A comprehensive review by Gasparini et al. summarized the role of serotonin in migraine pathogenesis, with low levels of serotonin, genetic polymorphisms, and triptan response in migraineurs being some of the topics explored [29]. Even decreased levels of tryptophan have been observed in migraine patients [26, 29].

In a meta-analysis of 10 studies, Schurks et al. reported no association between 5HTTLPR and migraine development. However, carriers of the S allele of 5HTTLPR have increased the risk of migraine development [23]. Moreover, research done by Lee et al. on 186 patients with depression found that 5HTTLPR positively correlated to depression in comparison to controls [24]. When it comes to the MID population, recent studies report no significant association between 5HTTLPR and migraine combined with depression [25].

Serotonin's role in depression has been well documented, and low serotonin levels have been the principal focus of depression therapy. Given that, studies have linked 5HT levels to migraine and depression, it is hypothesized that it plays a role in the development of MDD.

MTHFR Polymorphism

The methylenetetrahydrofolate reductase (MTHFR) gene is located on chromosome 1p36.3 and is responsible for the enzyme MTHFR, which plays a crucial role in folate and homocysteine metabolism. In particular, the C677T gene variant, which occurs due to a point mutation resulting in valine substitution for alanine (C677C), has been indicated in both migraine and depression [30-33].

Studies done by Samaan et al. and Rubino et al. have demonstrated the role of the MTHFR C677T gene variant in the development of migraine with aura [30, 31]. Raised homocysteine levels have been associated with migraine with aura development. This increase in homocysteine levels could lead to endothelial dysfunction and the development of cortical diffusion inhibition, thus contributing to migraine with aura [34, 35]. MTHFR C677T allele was also a significant predictor for increased homocysteine levels and migraine with aura [35].

A meta-analysis done by Jiang et al. on a total of 13 case-control studies, including 1895 depressed patients and 1913 controls, found that the T variant of MTHFR C677T gene polymorphism was significantly associated with an increased risk of depression in the Chinese population (T vs. C: OR = 1.52, 95% CI = 1.24-1.85) [32]. Similarly, research done by Kelly et al. observed that among 100 depressed patients and 89 controls, 70 in the depressed population were associated with the MTHFR C677T variant compared to 49 in the control group of non-depressed people (OR: 1.90) [33].

Since both migraine and depression are linked to the MTHFR C667T genetic variant, it may be speculated that it may also play a role in MID.

Structural Brain Changes

Several studies have shown that both migraine and depression share some unusual brain activity [36-38]. In China, a study was done by Yang et al. amongst 93 people divided into four groups: MID, migraine without depression, MDD, and healthy controls. Functional MRI (fMRI) scans were done to detect abnormal brain activity in all the groups. It was found that changes in the right paracentral lobule and right fusiform gyrus were specific for MID. In migraineurs, alterations in the left thalamus, medial orbital of superior frontal gyrus, and triangular part of inferior frontal gyrus were noted. Affective symptoms seen in migraine were associated with functional changes in the right paracentral lobule, left calcarine, and left dorsolateral superior frontal gyrus [38].

An imaging study was done by Ma et al. comparing brain scans (MRI) among patients with MID (n=10), migraine without depression (n=22), MDD (n=13), and a control group (n=27). The changes in intrinsic brain activity reflected the effect of depression in migraineurs. Combined migraine and depression affected the left medial prefrontal cortex, which has been shown in prior studies to be responsible for self-referential mental activity in migraineurs and increased amplitude of low-frequency fluctuation (ALFF) in MDD [36, 39-43]. They concluded that the abnormal medial prefrontal cortex might be responsible for the shared symptoms in migraine with comorbid depression. Another relevant finding was that those with MID had different developmental trajectories in the right thalamus and fusiform gyrus. It was also noted that the MID population had distinctly decreased thalamic activity compared to those without depression [36].

The above studies elicit the shared brain activity changes that may be responsible for MID.

Hormones

Both migraine and MDD are more common in women. Migraines are three times more common in women, while MDD is nearly twice as common in men [1, 44]. Ovarian hormones have various effects on the nervous system of a female. Progesterone activates the GABAergic system and modulates estrogen activity in the central nervous system. Both estrogen and progesterone affect the vascular endothelium and pain processing systems involved in the pathophysiology of migraine [45]. Furthermore, estrogen has been shown to have an overall antagonist effect on the serotonergic system, which has been associated with both migraine and depression [46]. In contrast, progesterone, in general, has a more positive impact on the serotonergic system by reducing the gene expression of monoamine oxidase, decreasing monoamine oxidase, and increasing serotonin levels, which improves both depression and migraine [45].

Shared Environmental Factors, Stress, and Obesity

It has been concluded that environmental factors contribute to the development of depression. Also, migraines have been known to be triggered during times of stress [47].

One of the conclusions of the study done by Yang et al. was that similar environmental factors could contribute to the development of both migraine and comorbid depression [15]. Research done by Schur et al. in 2009 showed shared heritability of MID and also reinforced the influence of gene-environment interactions in the etiology of migraine and depression [16].

Canadian prospective cohort research done by Swanson et al. in 2013 studied the contribution of stress to comorbid depression in migraine patients. Over 9000 Canadians were followed for eight years. They found that chronic stress can cause depression and chronic pain. Their results were significant for establishing the bidirectional relationship between migraine and depression, similar to previous studies. Interestingly, the significance was considerably attenuated and, in some cases, lost its significance once stressors were taken into account. Among the various stressors, chronic stress was the most substantial risk factor, implying that it may be a cause of chronic pain and depression [48].

In an earlier study done by Tietjen et al. in 2007, in 949 women with migraine, migraineurs with comorbid depression reported higher frequencies of physical or sexual abuse in childhood than those without depression. This demonstrated that women with MID have a higher prevalence of maltreatment in childhood [49].

Moreover, shared comorbidities like obesity could possibly affect the genetic associations between both these disorders [49]. Tietjen et al. showed that obesity was associated with current depression (OR = 1.86, 95% CI: 1.25 to 2.78) and obese migraineurs with depression were more likely to have higher headache frequency (OR = 4.16, 95% CI: 1.92 to 8.99) and headache-related disability (OR = 7.10, 95% CI: 2.69 to 18.77) compared to normal weight migraineurs without depression [50].

In an earlier study done by Tietjen et al. in 2007, in 949 women with migraine, migraineurs with comorbid depression reported higher frequencies of physical or sexual abuse in childhood than those without depression. This demonstrated that women with MID have a higher prevalence of maltreatment in childhood [49].

Moreover, shared comorbidities like obesity could possibly affect the genetic associations between both these disorders [49]. Tietjen et al. showed that obesity was associated with current depression (OR = 1.86, 95% CI: 1.25 to 2.78) and obese migraineurs with depression were more likely to have higher headache frequency (OR = 4.16, 95% CI: 1.92 to 8.99) and headache-related disability (OR = 7.10, 95% CI: 2.69 to 18.77) compared to normal-weight migraineurs without depression [50].

Clinical implications

**Figure 1:**
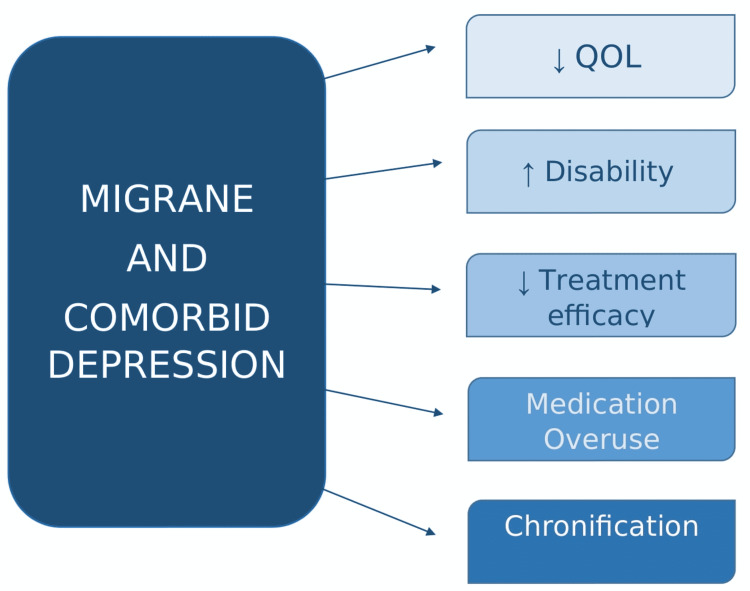
Summary of clinical implications of MID QOL: Quality of Life

Since the bivariate relationship between migraine and depression has been established, a further outlook on the implications of this association was warranted. Several types of research have highlighted the impact of depression on migraine disability, quality of life (QOL), medical costs, and medication overuse (Figure 1).

QOL and Migraine Disability

Patients with comorbid depression usually report more severe symptoms compared to those without depression. Studies have shown reports of more headache days, greater disability, and poorer QOL.

In a sample of about 4000 migraineurs, Jette et al. found that around 19% had a lifetime prevalence of MDD. Those with mental health disorders had a higher likelihood of poor outcomes. They reported increased two-week disability, restricted activities, poorer QOL, and less use of mental health care services [51].

A study done by Heckman et al. on 311 migraineurs in the US reported that those with comorbid depression had comparable headache days and headache severity to those without psychiatric conditions; however, they reported more significant disability and poorer QOL compared to those without psychiatric conditions [52]. The earliest research studying the effect of comorbid psychiatric disorders, in 2005 done by Lanteri-Minet et al., observed that the presence of depression and anxiety in a population of 1957 migraineurs significantly affected migraine disability and quality of life negatively. They found that perceived treatment efficacy and satisfaction with treatment were lower in those with comorbid depression [53].

A nation-based population study done by Saunders et al. found that 83% of migraineurs and 79% of those with other severe types of headache were likely to have associated comorbidities, which could be a physical condition, chronic pain condition, or a mental condition. They elicited that migraine disability was indeed more severe in those with comorbid depression in comparison to migraineurs without depression [54].

Similar to the studies reviewed above, Pradeep et al. derived the same conclusion in a study conducted on 108 migraineurs. They found that co-existing anxiety or depression was responsible for a greater magnitude of migraine-related disability and lower QOL [55].

All this research drive home the effect of depression on migraine.

Chronification of Migraine

The prevalence of chronic migraine ranges from 1.4% to 2.2% [3]. Chronic migraines not only cause poorer QOL compared to EM, but it also contributes to the development of further depression [56].

Depression has been discovered to be a contributory factor in the progression of EM to CM. As evidenced by the longitudinal population-based study done by Ashina et al., depression was a significant predictor of CM onset (OR = 1.65, 95% CI: 1.12-2.45) and predated CM occurrence. Furthermore, this association was dose-dependent as those with moderate, moderately severe, and severe depression were at an increased risk of CM development compared to those with no or mild depression (OR = 1.77, OR = 2.35, OR = 2.53, respectively). In conclusion, the presence of depression in EM for just one year predicted the onset of new CM in the next year [57]. This association, however, may also be explained by common risk factors shared by migraine and depression, such as stress [58]. Also, chronic headache and debility themselves promote the development of depression and a lower rate of remission, forming an endless cycle that worsens both these disorders [58, 59].

Medication Overuse

Medication overuse headache (MOH) is defined as a headache that occurs more than 15 days a month for three months or more in people with pre-existing headache disorders who overuse acute medical treatment. It usually stops after the discontinuation of the medication [60]. Around 70% of people with chronic daily headaches are estimated to have medication-overuse headaches [61]. As per the International Classification of headache disorders, the chief risk factor of migraine chronification is acute migraine medication overuse [60]. In a longitudinal population-based cohort study done by Hagen et al. in over 25,000 patients over 11 years, it was found that those with anxiety or depressive symptoms (Hospital Anxiety and Depression Scale score ≥ 11) were about five times more likely to develop MOH [62]. As depression is linked to greater severity and debility in EM, these individuals likely overindulge in abortive medications, which leads to medication overuse headache [63]. This further complicates the management of migraine with depression.

Treatment modalities

Efficient management of MID becomes imperative, given its impact on the clinical course and progression. For that purpose, the commonalities between migraine and depression were targeted, such as the use of antidepressants to treat both simultaneously. Several alternative drugs and treatment methods have been sought to answer this conundrum.

Antidepressants

Various classes of antidepressants - like Selective Serotonin Reuptake Inhibitors (SSRI), Selective Serotonin and Norepinephrine Inhibitors (SNRI), and Tricyclic Antidepressants (TCA) - were investigated.

Keskinbora and Aydinli published a three-month-long study in 2008 dealing with three groups of patients: the first group was given only topiramate throughout the trial (n=20), the second group was given only amitriptyline (n=22), and the last group was given a combination of topiramate and amitriptyline (n=21). The study was carried out on non-depressed migraineurs in Turkey and concluded that the combination of both medications was possibly beneficial in patients who are being treated for migraines with comorbid depression [64].

Landy et al. published a randomized control trial (RCT) in 1999 on the effect of sertraline as a prophylactic agent on migraines. Results of the eight-week study concluded that it was less effective compared to conventional prophylaxis in migraine treatment. However, the authors advocated its possible use in cases of chronic headaches with comorbid depression [65].

Once the efficacy of select antidepressants in migraine was established, other studies were undertaken to assess their usefulness in MID. A study done by Rampello et al. in 2004 over 16 weeks in Italy demonstrated the efficacy of antidepressants in the management of MID. They showed that amitriptyline was more effective at preventing tension-type headaches (TTH) and MID compared to citalopram. Interestingly, those unresponsive to monotherapy with either antidepressant showed significant improvement in depression, TTH, and migraine. Overall, depression improved equally with amitriptyline and citalopram monotherapy [66].

Another SSRI, sertraline, was shown to have improved QOL in MID in a study done by Macgregor et al. in 2011. Sertraline was administered to those with MID over 16 weeks. Depression and QOL were assessed at baseline and every four weeks during the study period. Although initially, the migraine risk increased during the first few weeks, this risk was resolved with continued use of sertraline. Overall, QOL and depression improved significantly compared to baseline, and no symptoms of serotonin toxicity were observed [67].

Curone et al. conducted a study on a population of 50 migraineurs with CM and MOH along with concurrent depression. After the first month, which was established as the baseline, they were assessed on the efficacy of duloxetine treatment after a period of three months. Hamilton Depression Rating Scale (HDRS) and Migraine Disability Assessment Scale (MIDAS) were completed at baseline and at the 12-week follow-up. Their findings suggested significant improvement in CM due to MOH in depressed migraineurs [68].

Volpe did an eight-week-long study on patients with depression and primary chronic headache (CM, chronic tension headache, or both). Similar to the previous study, duloxetine showed considerable efficacy in improving both depressive symptoms and migraine-associated debility (Table 2) [69].

**Table 2:** Summary of studies assessing the efficacy of SSRIs/SNRIs/TCAs in MID QOL: Quality of Life; TTH: Tension-type headache; HDRS: Hamilton Depression Rating Scale; PHQ-9: Patient Health Questionnaire (PHQ)-9; Q-LES-Q: The Quality of Life Enjoyment and Satisfaction Questionnaire; SF 12: Short Form Health Survey; MIDAS: Migraine disability assessment scale; OCD: Obsessive-compulsive disorder; CM: Chronic migraine; CTTH: Chronic tension-type headache; MADRS: Montgomery-Asberg Depression Rating Scale; VAS: Visual analog pain scale; WHOQoL-BREF: World Health Organization Quality of Life scale; PGIC: Patient Global Impression of Change; HIT-6 scores: 6-item Headache Impact Test; SSRI: Selective Serotonin Reuptake Inhibitors; SNRI: Selective Serotonin and Norepinephrine Inhibitors; TCA: Tricyclic Antidepressants.

References	Design	Sample size	Groups	Sample population	Headache Index/QOL	Depression Index
Rampello et al. (2004) [66]	Randomized, open, community-based study done over 16 weeks	88	Amitriptyline (44) Citalopram (44) Amitriptyline + Citalopram in those not responsive to amitriptyline monotherapy (8) Amitriptyline + Citalopram in those not responsive to citalopram monotherapy (21)	Patients with: 1- ≥10 TTH/ month, 2- migraine without aura: 3-5 episodes/month, 3- HDRS score between 20 and 35, 4- Patients had not consumed triptans and had not been on prophylactic treatment before the study.	Number of migraine attacks per month and number of days with TTH per month	HDRS score
MacGregor et al. (2011) [67]	16-week open-label trial	51	Use of sertraline 50mg in 35 people on triptans	Women aged between 18 and 65 years, with migraine (with or without aura).	Q-LES-Q, SF-12 mental, SF physical	PHQ-9
Curone et al. (2013) [68]	Open label prospective independent study over 12 weeks	50	Duloxetine 30mg in the first week and 60mg in the following weeks	50 patients aged 20-65 years having chronic migraine with depression and medication overuse	MIDAS	HDRS score
Volpe (2008) [69]	Open trial study over 8 weeks	30	Duloxetine 60mg	Adults having MDD with concurrent chronic headache (CM, CTTH, both) in Brazil	VAS WHOQoL-BREF	MADRS

Alternative Treatments

OnabotulinumtoxinA (OBTA) is one of the neurotoxins produced by Clostridium botulinum. Its usefulness in headache disorders was discovered by chance in 1998. Subsequently, it was studied extensively in the prevention of various headaches [70]. Other than Calcitonin Gene-Related Peptide (CGRP) antibodies, it is the only FDA-approved treatment for the prevention of CM [71].

A study done on 32 patients by Boudreau et al. showed that administration of OBTA in patients with chronic migraine and comorbid depression effectively reduced the attacks, severity, and disability related to migraine which led to improvement in depression [72]. Similar results were found in research conducted by Zhang et al. on 30 patients who reported alleviated severity and frequency of headaches and improvement in depression [73]. However, other studies suggest that the presence of comorbid depression reduces the efficacy of OBTA [74].

Cognitive-behavioral therapy (CBT) is a form of psychological therapy that has been demonstrated to be effective in a range of disorders such as MDD, anxiety, substance abuse, eating disorders, and severe mental illnesses. In recent years, CBT has been drawing attention as an adjunct, if not an alternative treatment for migraines [75]. There is evidence that CBT can help alleviate migraine symptoms and may also decrease the frequency of headache episodes [7, 76].

An RCT done by Martin et al. compared the efficacy of CBT against standard therapy in 66 patients suffering from migraine/tension headache with MDD. 42% of the CBT receiving group demonstrated a significant reduction in headaches along with decreases in depression scores [77]. In contrast, a meta-analysis by Sharpe et al. consisting of 21 RCTs of 2482 patients with migraine was unable to garner any significant results of psychological therapy in migraine prophylaxis [78].

As MDD is often treated with CBT, it may be speculated that CBT may prove efficacious in managing migraine with comorbid depression.

Finally, vagus neurostimulation has been proposed as a possible treatment option as it has displayed efficacy in both migraine and depression independently [79, 80]. The vagus nerve is a mixed motor and sensory nerve. Its visceromotor efferent fibers originate from the dorsal motor nucleus of the medulla, whereas its afferent counterpart terminates at the nucleus tractus solitarius (NTS). The NTS projects to areas of the brain implicated in depression pathogenesis, such as the dorsal raphe nucleus and locus ceruleus, which then project to limbic forebrain areas [81]. Vagal nerve stimulations (VNS) usually involve the implantation of a generator stimulator around the left vagus nerve, although noninvasive stimulation has also shown some efficacy [82].

In a case series conducted by Cecchini et al., VNS treatment administered over three months in four patients with drug-refractory CM and depression improved migraine and depressive symptoms in only two of the patients. The study concluded that long-term follow-up was required to evaluate the efficacy of VNS in those who had limited improvement [83].

Monoclonal Antibodies

Although studies have been more focused on conventional antidepressants as a possible treatment, newer research done in the last couple of years has unveiled a novel therapy with very promising results. Calcitonin gene-related peptide (CGRP) has been heavily implicated in the pathophysiology of migraine, and drugs targeting it have recently been developed [84]. Fremanezumab, a fully-humanized monoclonal antibody that targets CGRP, was recently approved by the FDA and European Medical Association (EMA) for the preventive treatment of migraine [85]. It was shown to be effective in both episodic and chronic migraine [86-88].

On evaluating the efficacy in migraineurs with comorbid depression, fremanezumab improved symptoms significantly. Lipton et al. conducted post hoc analyses on the migraine with comorbid depression subpopulation (n=219) in the original HALO study [ClinicalTrials.gov Identifiers: NCT02621931, NCT02629861]. These individuals had moderate to severe depression (PHQ-9 score ≥10) at baseline and were analyzed on the number of headache days of minimum moderate severity occurring per month; monthly migraine days; Patient Global Impression of Change (PGIC); 6-item Headache Impact Test (HIT-6) scores; and depression. Over 12 months, one-third of the group received fremanezumab quarterly, the second-third received fremanezumab monthly, and the remaining were administered a placebo. In the quarterly and monthly groups, 51% and 55% had moderate depression. Moderate - severe depression was seen in 36% and 35%, respectively, and 12% and 10% had severe depression. The results concluded that fremanezumab was efficacious in reducing headache days and improving depression compared to the baseline number of headache days was reduced by about 7-8 days, and there was a decrease of around 10 points in the PHQ - 9 score [89].

So far, only this one study has been done to evaluate fremanezumab in MID. However, encouraged by the results, at least one more is currently ongoing [ClinicalTrials.gov Identifier: NCT04041284]. Similarly, another CGRP monoclonal antibody, galcanezumab, has shown some effectiveness in MID [90].

Treatment Adjustment

Triptans have been one of the primary treatment options for abortion of migraine episodes. Similarly, SSRIs and SNRIs are usually the first line in depression management. Therefore, in MID, combined treatment with triptans and SSRI/SNRI raised concerns regarding drug interactions.

In 2006, the US FDA issued an alert on the combined use of SSRIs or SNRIs with triptan medications as potentially life-threatening. Reviews done by Evans et al. in 2007 and 2010 of the 29 cases provided by the FDA as a basis for the alert used both the Sternbach criteria [91] and Hunter criteria for serotonin syndrome diagnosis. They found that only seven (first review) and 10 (second review) met the Sternbach criteria, and none met the Hunter criteria [92, 93].

A 2018 study by Orlova et al. reviewed over 47,000 unique patients that were prescribed triptans from January 2001 to December 2014, out of which over 19,000 were prescribed antidepressants, with a total of 30,928 person-years of exposure. Serotonin syndrome incidence was 0 to 4 per 10,000 person-years of exposure in patients’ co-prescribed triptans and SSRIs/SNRIs, and only 17 patients were suspected of possible serotonin syndrome (2.3 patients per 10,000 person-years of exposure; 95% CI, 0.6-3.9). This indicated that the risk of serotonin syndrome in co-prescription of triptans and antidepressants was quite low [94].

Another study by MacGregor et al., conducted in 2011, found that no cases of serotonin syndrome developed in a group of 35 patients when combining SSRIs and SNRIs with triptans. They concluded that an absolute contraindication to co-prescription should be reconsidered as it may cause mismanagement of migraine with depression [67].

Although these studies reported a very low incidence of serotonin syndrome on SSRIs/SNRIs and triptans co-prescription, cases fitting this exact picture are still being reported. Jin and Stokes in 2021, presented a case of serotonin syndrome in a 30-year-old woman who was prescribed sumatriptan while on fluvoxamine for long-standing depression. This case demonstrates that despite the low incidence of drug interactions between these drug classes, the possibility of serotonin syndrome must not be dismissed [95]. Therefore, caution must be advised when prescribing these drugs concurrently.

## Conclusions

The studies reviewed in this article highlight the shared pathogenicity of migraine and depression and the obstacles faced in managing this condition. This is significant since the clinical impact is quite severe, leading to treatment resistance and progression of the disorder, to the point where it may even be classed as a separate illness rather than a subtype of migraine. The decreased quality of life and medication overuse contributes to an increased financial burden. As evident from our review of relevant research, solutions to tackle this problem have been few and far between, with various methods implemented but none offering a clear answer. Once accurately diagnosed, steps can be taken to alleviate the condition by managing both the migraine and depression components. Although one drug to target both simultaneously (such as the use of antidepressants) would be ideal; unfortunately, a magic pill is yet to be discovered. The advent of anti-CGRP antibodies offers hope; however, it still needs extensive testing before being declared effective. In the meantime, a combination of methods may be used, and the focus should not solely be on medications but also on external factors such as stress relief and CBT. While prescribing abortive drugs and antidepressants concurrently, the possibility of serotonin syndrome should be borne in mind and the patient should be carefully monitored. We believe that there is still a way to go before the approach to migraine with comorbid depression is well defined. Therefore, further research must be undertaken to shed light on this matter.
